# Engineered Exosomes Biopotentiated Hydrogel Promote Hair Follicle Growth via Reprogramming the Perifollicular Microenvironment

**DOI:** 10.3390/pharmaceutics16070935

**Published:** 2024-07-13

**Authors:** Hairui Zhang, Jiali Yao, Qianyang Jiang, Yurou Shi, Weihong Ge, Xiaoling Xu

**Affiliations:** 1 School of Pharmaceutical Sciences, Zhejiang Chinese Medical University, Hangzhou 310053, China; hairuizhang@zcmu.edu.cn (H.Z.); 15825565999@163.com (J.Y.); 2 Key Laboratory of Artificial Organs and Computational Medicine in Zhejiang Province, Shulan International Medical College, Zhejiang Shuren University, Hangzhou 310015, China; 202211002122@stu.zjsru.edu.cn (Q.J.); shiyr8-30@foxmail.com (Y.S.)

**Keywords:** androgenetic alopecia, flexible liposomes, engineered exosomes, hair follicle, reprogram the perifollicular microenvironment

## Abstract

Androgenetic alopecia (AGA) is a highly prevalent condition in contemporary society. The conventional treatment of minoxidil tincture is hindered by issues such as skin irritation caused by ethanol, non-specific accumulation in hair follicles, and short retention due to its liquid form. Herein, we have developed a novel minoxidil-incorporated engineered exosomes biopotentiated hydrogel (Gel@MNs) that has the capability to modulate the perifollicular microenvironment for the treatment of AGA. Leveraging the exceptional skin penetration abilities of flexible liposomes and the targeting properties of exosomes, the encapsulated minoxidil can be effectively delivered to the hair follicles. In comparison to free minoxidil, Gel@MNs demonstrated accelerated hair regeneration in an AGA mouse model without causing significant skin irritation. This was evidenced by an increase in both the number and size of hair follicles within the dermal layer, enhanced capillary formation surrounding the follicles, and the regulation of the transition of hair follicle cells from the telogen phase to the anagen growth phase. Therefore, this safe and microenvironment-modifying hybrid exosome-embedded hydrogel shows promising potential for clinical treatment of AGA.

## 1. Introduction

Androgenic alopecia (AGA) is the most common hair loss disorder [1,2,3], affecting 80% of men and 50% of women throughout their lives [4]. And studies have shown that the prevalence of AGA in men is as high as 30% by the age of 30, as high as 50% by the age of 50, and as high as 80% by the age of 70, and the incidence increases with age [5,6,7]. It is worth noting that AGA is a progressive alopecia. Without timely intervention, the area of hair loss will gradually increase with the progression of the disease, and the hair follicles will eventually enter an irreversible miniaturization state [8,9]. However, the current treatment for AGA is very limited, mainly including drugs, surgery, and other supportive therapy [10].

The existing topical drug treatments focus on minoxidil tincture, which is formulated as a solution containing ethanol, propylene glycol, and water [11]. Research indicates that approximately 60% of users have experienced significant clinical improvements [12,13]. However, there were some limitations. (1) Serval side effects. The addition of ethanol enhances the transdermal efficiency of minoxidil, a fat-soluble drug, but it also inevitably results in skin irritation. Some individuals may experience discomfort such as scalp irritation, dryness, and itching during treatment [14,15,16]. (2) Non-specific distribution. Conventional minoxidil lacks preferential accumulation in hair follicles, leading to suboptimal efficacy in improving the microenvironment within the hair follicles. (3) Reduced therapeutic outcomes. The liquid nature of the formulation poses challenges in maintaining contact between the drug and the target area, such as the scalp or hair follicles. This can result in reduced absorption and compromised therapeutic effectiveness [17,18].

The advent of liposome-based nanocarriers offers promising prospects for delivering drugs to targeted areas without the need for ethanol, which holds significant potential for hair loss treatments [19,20,21]. A novel advancement in this field is the development of flexible liposomes, a new generation of liposomes that incorporate edge activators like Tween 80 to enhance their flexibility and deformability [22,23]. This heightened deformability allows the liposomes to navigate through narrow intercellular spaces, thereby enhancing their capacity to penetrate the layers of the skin [24]. Nevertheless, flexible liposomes are currently deficient in their ability to achieve targeted delivery specifically to the hair follicles.

Cell-derived exosomes with homing abilities offer a promising avenue for delivering drugs specifically to the hair follicles. Research has highlighted the potential of exosomes derived from follicular constituent cells, such as DPC-derived exosomes, in modulating the biological processes of hair follicles by releasing a variety of growth factors and upregulating specific genes. For instance, the upregulation of miR-218-5p in exosomes can directly target SFRP2, leading to the upregulation of the WNT/β-catenin pathway and stimulating hair growth [25]. Furthermore, DPC-derived exosomes have been shown to significantly enhance wound healing through KLF4/VEGFA-driven angiogenesis, which could play a crucial role in the Mogadishu effective treatment of hair loss with DPC-derived exosomes [26]. Therefore, exploring the fusion of flexible liposomes and exosomes could potentially enable targeted drug delivery to the hair follicles while minimizing side effects. However, engineered exosomes still retain the characteristic of being a solution, which is prone to leakage. 

The elevated viscosity of hydrogels provides superior adhesion and cohesion, enabling the gel to adhere to the target surface for an extended duration, thereby prolonging contact time and augmenting drug absorption [27,28,29]. Notably, carbomer-based polymers are frequently employed in the composition of hydrogels for managing skin-related conditions and have garnered significant attention [30]. Carbomer gel is a water-based gel that typically does not contain organic solvents or irritating ingredients, resulting in minimal skin irritation and positioning it as a promising embedding material for engineered exosomes [31,32].

Herein, we prepared minoxidil-incorporated flexible liposomes and fused them with mDPC-derived exosomes. Subsequently, the engineered exosomes were mixed with the carbomer-based gel to obtain hybrid nanoparticle biopotentiated hydrogels for the treatment of a mouse AGA model. Our findings demonstrated a significant increase in both the number and size of hair follicles within the dermal layer of the skin, enhanced capillary formation surrounding the follicles, and modulation of the transition of hair follicle cells from the telogen phase to the anagen growth phase. Collectively, this safe and microenvironment-altering hybrid exosome-embedded hydrogel reveals significant potential for the clinical treatment of AGA.

## 2. Materials and Methods

### 2.1. Extraction and Validation of Primary Mouse Dermal Papilla Cells

The primary mouse dermal papilla cells were extracted using a mixture of collagenase and a neutral protease digestion method. In detail, the newborn mice were immersed in a 75% ethanol (Boer, B601764, Shanghai, China) beaker to death, moved to the ultra-clean bench, fixed on the back, and the complete skin at the beard was cut off; after soaking in PBS containing double antibody for 5 min, the subcutaneous fat and connective tissue were carefully stripped, and a single hair follicle tissue was clipped from the dermal surface with microscopic tweezers. The hair was removed, the hair follicle tissue was retained, and the tail red dermal papilla tissue was cut and collected. Put the tissue into a centrifuge tube, add digestive fluid (collagenase I, neutral protease II, DMEM complete medium preparation) (Procell, Wuhan, China), enable digestion at 37 °C for 16–18 h; take out the centrifuge tube, and use a pipette to repeatedly blow the tissue to the liquid turbidity; the digestive fluid was filtered with 100 mesh and 200 mesh cell sieves, and the filtrate was collected. The filtrate was centrifuged at 300× *g* for 5 min, the supernatant was discarded, and the cell precipitate was retained.

We validated the primary mouse dermal papilla cells using immunofluorescence staining for the surface marker Vimentin. First, the adherent cells were fixed with 4% paraformaldehyde (Beyotime, P0099, Shanghai, China) for 15 min, washed with PBS, passed through with 0.5% TritonX-100 (Beyotime, P0106, Shanghai, China) at room temperature for 20 min, and blocked with 5% goat serum (Beyotime, C0265, Shanghai, China) for 30 min. Then, Rabbit anti-Vimentin (abcam, ab92547, Cambridge, UK) was added for overnight incubation at 4 °C, and cells were incubated with Cy3-goat anti-rabbit IgG (abcam, ab6939, Cambridge, UK) at room temperature for 30 min. Finally, DAPI (Beyotime, C1002, Shanghai, China) was added to incubate for 5 min in the dark, and the specimens were stained. The images were observed with an AXIO Observe Inverted Fluorescence Microscope (Zeiss, Oberkochen, Germany).

### 2.2. Extraction and Validation of Extracellular Vesicles from mDPCs

The collected cell culture medium was centrifuged at 1500× *g* for 20 min to remove cells and cell debris, at 17,000× *g* for 15 min to remove organelles, and at 110,000× *g* for 80 min [33]. A small amount of white precipitate was observed at the bottom, and fresh extracellular vesicles were obtained after resuspension with PBS. The whole process was carried out at 4 °C.

Extracellular vesicles-related markers were detected via Western blot [34,35,36]. The extracted extracellular vesicle solution was added to a protein lysate (Beyotime, P0013B, Shanghai, China) containing a protease inhibitor (Beyotime, P1005, Shanghai, China) and lysed at 4 °C for 30 min. The supernatant was centrifuged at 13,000× *g* for 10 min to obtain the supernatant for BCA protein concentration detection (Solarbio, PC0020, Beijing, China). After adjusting the protein concentration of the sample, the protein loading buffer (Beyotime, P0015, Shanghai, China) was added, and the protein was denatured using a metal bath at 95 °C for 10 min. Equal volumes of the samples were separated with 10% SDS-polyacrylamide gel electrophoresis (Beyotime, P0901M, Shanghai, China). The separated proteins were then transferred to a polyvinylidene difluoride membrane (Millipore, IEVH85R, New Bedford, MA, USA) and blocked with 5% skim milk (BD, 232100, Becton Drive Franklin Lakes, NJ, USA). The membranes were then incubated overnight with Rabbit anti-CD63 (abcam, ab134045, Cambridge, UK), Rabbit anti-Tsg101 (abcam, ab125011, Cambridge, UK), and mouse anti-Alix (abcam, Alix, Cambridge, UK), followed by incubation with Goat anti-Rabbit IgG (CST, 7074S, Danvers, MA, USA) and horse anti-mouse IgG (CST, 7076S, Danvers, MA, USA) conjugated to horseradish peroxidase. Electrochemiluminescence chromogenic substrate was added to visualize the target bands using a ChemiDoc imaging system (Bio-Rad, Shanghai, China).

### 2.3. Preparation of Gel@MNs

First, NHS-PEG-Lio@M was prepared using the thin film dispersion method. In simple terms, 1 mg of DSPE-PEG_2000_-NHS (Shanghai ToYangBio, P006005-2K, Shanghai, China), 14 mg of Lecithin (Macklin, L812368, Shanghai, China), 4 μL of Tween-80 (Macklin, T796772, Shanghai, China), and 5 mg of minoxidil (Aladdin, M158689, Shanghai, China) were weighed and dissolved in 5 mL of anhydrous ethanol (Aladdin, E118433, Shanghai, China). The solution was then sonicated to facilitate dissolution. Next, the solvent was evaporated using a rotary evaporator at 40 °C to remove the anhydrous ethanol. Subsequently, 1 mL of distilled water was added to the solution for hydration, followed by membrane removal using a water bath sonicator. Lastly, the solution was subjected to sonication using a cell disruptor probe at 600 W for 10 min to achieve a uniform distribution of the nano solution. Centrifuge the solution using a 30 kDa ultrafiltration membrane (Millipore, ACK5030NT, New Bedford, MA, USA) at 5000 rpm for 15 min. The solution collected at the bottom will be used for subsequent analysis. Resuspend the solution from the upper layer to its original volume.

Next, mix NHS-PEG-Lio@M with EVs from mDPCs and use Extruder (Avanti, 610020, Birmingham, AL, USA) to push the mixture back and forth 20 times to achieve uniform fusion into MNs.

To synthesize Gel@MNs, Carbopol 940 (1%) (Macklin, C832684, Shanghai, China) is added to purified water and allowed to swell for 24 h. A water solution containing glycerol (10%) (Macklin, G81057, Shanghai, China 5), EDTA (0.1%) (Macklin, E809068, Shanghai, China), and hydroxybenzoate (0.1%) (Macklin, E808808, Shanghai, China) is then added. Triethanolamine (0.8%) (Sigma, 471283, Shanghai, China) is added to adjust the pH to suit the skin environment, resulting in the formation of a blank gel. Subsequently, the blank gel and the nano solution of MNs are mixed in a volume ratio of 3:1 to obtain the desired drug.

### 2.4. Characterization of Gel@MNs

For examining the morphology of the nanoparticles, the size and Zeta potential of the nanoparticles were measured using Dynamic Light Scattering (DLS, Zetasizer Ultra, Malvern, Shanghai, China). The nanoparticle solution was drop-cast onto the carbon-coated copper grids, which were left to air-dry at room temperature. The morphology of the nanoparticles was observed using a transmission electron microscope (TEM) (JEOL, JEM-1230, Tokyo, Japan). Additionally, we obtained freeze-dried Gel@MNs for Scanning Electron Microscopy (SEM) (HITACHI, TM4000Plus, Ibarakiken, Japan) analysis.

### 2.5. Measurement of Drug Loading Capacity and Encapsulation Efficiency

In the preparation of NHS-PEG-Lio@M, the solution collected at the bottom was collected for UV analysis (Multiskan SkyHigh full-wavelength microplate reader, Thermo Scientific™, A51119500C, Waltham, MA, USA). Simply put, the standard solution of minoxidil was dissolved in anhydrous ethanol, and the absorption wavelength of 200 to 400 nm was detected in the UV detector. The standard curve of minoxidil was obtained according to the OD value at the maximum absorption wavelength of the standard at different concentrations, and the filtrate was diluted to a certain multiple to detect the concentration of minoxidil in the sample.

Drug loading (DL%) and encapsulation efficiency (EE%) were calculated using the following formulas:

$$DL\%=\frac{weight\;of\;feeding\;Minoxidil-drug\;concentration\times volume}{weight\;of\;NHS-PEG-Lio@M}\times 100\%$$

$$EE\%=\frac{weight\;of\;feeding\;Minoxidil-drug\;concentration\times volume}{weight\;of\;feeding\;Minoxidil}\times 100\%$$

### 2.6. Cell Viability Assay

L929 and Raw 264.7 cells were seeded at a density of 8 × 10^3^ cells per well in a 96-well plate overnight and incubated with PBS, 5 μg/mL minoxidil, 20 μg/mL Ns, and 25 μg/mL MNs for 24 h. Then, the medium was discarded and serum-free medium containing 10 μL of CCK8 solution (Cell Counting Kit-8, Beyotime, C0038) was added into each well, followed by incubation at 37 °C for 2 h. The absorbance at 450 nm was measured using a Multiskan SkyHigh full-wavelength microplate reader (Thermo Scientific™, A51119500C).

### 2.7. Detection of Skin Irritation of Gel@MNs

The back of Wistar rats was depilated and the area was about 2 cm in diameter. The back of the rats was ground with sandpaper, and the broken skin model was constructed with mild bleeding. In the following week, 5mg of minoxidil and 50 mg of Gel@MNs was applied daily and the skin recovery was photographed. The rat skin was quantified via the constructed skin redness and swelling score standard.

### 2.8. In Vivo Studies of the Gel@MNs for AGA Treatment

The hair regeneration capabilities of Gel@MNs were evaluated in male C57BL/6 mice (6 weeks old). A 1 cm^2^ area (1 cm × 1 cm) of the hair from the dorsal portion of the 7-week-old mice in the telogen phase was gently shaved with an electric hair clipper and depilated with hair removing cream. The mice were randomly divided into five groups: the model group, the minoxidil group, the Gel group (Blank Gel), the Gel@Ns group (minoxidil-free liposomes and EVs in Gel), and the Gel@MNs group (minoxidil liposomes and EVs in Gel). The establishment of the AGA mouse model is constructed. In brief, the testosterone (Solarbio, T8600) solution (0.2%, *w*/*v*) was prepared in ethanol solution (50%, *v*/*v*). Mice in all groups were topically applied with testosterone solution (0.1 mL/cm^2^) on the depilated area once a day for 28 consecutive days. Simultaneously, with the application of testosterone, Gel, Gel@Ns, and Gel@MNs were applied every day within 13 days after depilation. Additionally, 5% minoxidil was used as a positive control and was applied topically (0.1 mL/cm^2^) once a day until day 13. Digital pictures of skin and hair were taken. At the end of the test cycle, the mice were weighed and sacrificed, blood was collected, and tissue of the heart, liver, spleen, lung, and kidney was obtained.

### 2.9. Histological Staining of Skin Samples

Animals were sacrificed on day 28 for hematoxylin-eosin (H&E) staining and detection of VEGF levels as well as CD31 levels in the depilated area.

For paraffin sections, skin samples were fixed in formaldehyde (4%, *w*/*v*). Following sequential dehydration, tissues were embedded in paraffin, and sections of 3–4 μm thickness were obtained. Following deparaffination and rehydration, the sections were then stained with hematoxylin for 5 min and eosin for 1 min (Beyotime, C0105M). H&E slides were imaged with a slide scanner (Ningbo Shunyu, Ningbo, China). Among them, the thickness of the epidermis in the slice was quantitatively analyzed with Image J software (Version-lmage J 1.53t).

For immunofluorescence evaluations, deparaffinized and rehydrated sections of tissues were further incubated with Rabbit anti-CD31 (abcam, ab182981, Cambridge, UK) and Rabbit anti-VEGF (abcam, ab32152, Cambridge, UK) antibodies, followed by incubation with the corresponding fluorescence-labeled secondary antibodies and cell nuclear staining with DAPI. All immunohistochemical slides were photographed on a slide scanner. The fluorescence intensities of the stained markers were quantified using the Image J software (1.3t, NIH, Bethesda, MD, USA).

### 2.10. Allergic Detection of Skin Tissue

On day 28, the animals were euthanized, and a portion of the skin tissue was collected. Pre-chilled PBS was added, and the tissue was homogenized for 5 min using a tissue homogenizer at 4 °C. The homogenate was centrifuged at 5000 rpm for 20 min, and the supernatant was collected for IgE ELISA detection (MEIMIAN, MM-0056M2, Yancheng, China).

### 2.11. Examination of Tissue Toxicity

The major organs were collected, fixed, dehydrated, and paraffin-embedded routinely, and the prepared sections were then stained with H&E for histological examination.

Murine blood was collected in the anti-coagulant tubes containing sodium heparin. Blood was analyzed for HGB, PLT, RBC, WBC, NEU, LYM, MON, and EOS indexes using an automated hematology analyzer (TECOM, TEK8500-VET, Neufahrn, Germany).

### 2.12. RNA-seq of Mice Skin

RNAs were extracted from the skin of mice using TRIzol (Beyotime, R0016, Shanghai, China). Nanodrop 2000 (Thermo Fisher, Waltham, MA, USA) was used for measuring the concentration of extracted nucleic acids. Agilent 2100, LabChip GX were used for assessing the integrity of the nucleic acids. RNA sequencing libraries were prepared from 2 μg total RNA using the VAHTS Universal V6 RNA-seq Library Prep kit (Illumina, NR604-02, San Diego, CA, USA), which allows for strand-specific sequencing.

The first step of polyA selection using VAHTSTM DNA clean beads (N411-03) was performed to allow sequencing of polyadenylated transcripts. After fragmentation, cDNA synthesis was performed, and the resulting fragments were used for dA-tailing followed by ligation of TruSeq indexed adapters (Illumina, 20020492, San Diego, CA, USA). Subsequently, polymerase chain reaction amplification was performed to generate the final barcoded cDNA libraries. Sequencing was carried out on a NovaSeq 6000 instrument from Illumina based on a 2 × 100 cycles mode (paired-end reads, 100 bases).

Fastp software (Version-V0.20.1) was used to remove the junctions and low-quality sequences from the sequencing data, and CleanData was obtained after processing. The CleanData was aligned to the genome using HISAT2 (Version-hisat2-2.0.4) to obtain a bam file. The gene or transcript was initially assembled using StringTie software (Version-stringtie-1.3.4d. Linux_x86_64). The initial assembly results of all samples were merged, and the gffcompare software (Version-0.9.8. Linux_x86_64) was used to detect the comparison between the transcript and the reference annotation to obtain the final assembly annotation results. The ballgown package was used to provide file input for FPKM quantification, and DEseq2 (Version-1.260) was used to analyze the significant differences between samples. The genes with FoldChange > 2-times and *p*-value < 0.01 were defined as differential genes, and GO, KEGG, and KEGG analyses were performed.

### 2.13. Statistical Analysis

ANOVA and *t* test were employed for statistical analysis of differences between experimental group means. All statistical evaluations were performed using GraphPad Prism version 8 software. ns, nonsignificant (*p* > 0.05); * *p* < 0.05, ** *p* < 0.01, and *** *p* < 0.001.

## 3. Results

### 3.1. Fabrication and Characterization of the Gel@MNs System

**Figure 1:**
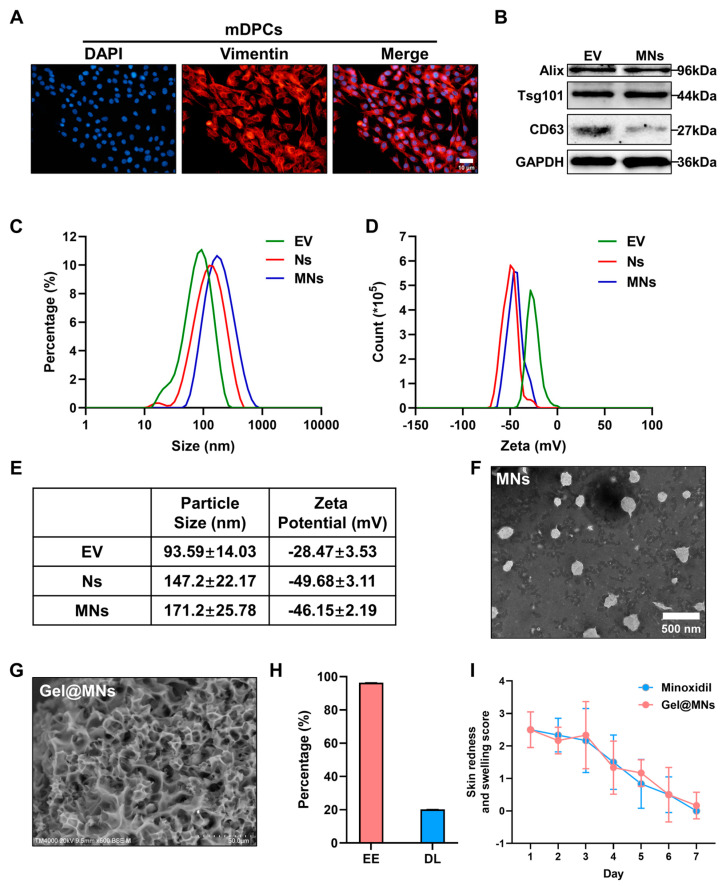
Characterization of Gel@MNs. (**A**) Immunofluorescence staining of Vimentin in mouse dermal papilla cells (scale bar = 10 μm). (**B**) Detection of exosome markers (CD63, Tsg101, and Alix) in the same amount (20 μg protein) of extracellular vesicles and MNs via Western blot. (**C**) Hydrodynamic size distribution of MNs, Ns, and EV via DLS. (**D**) Zeta potential of MNs, Ns, and EV via DLS. (**E**) Evaluation of average particle size and zeta potential of MNs, Ns, and EVs. (**F**) TEM images of MNs (scale bar = 500 nm). (**G**) SEM images of Gel@MNs (scale bar = 50 μm). (**H**) The encapsulation efficiency (EE) and drug loading (DL) of minoxidil in Gel@MNs. (**I**) Evaluation of skin redness and swelling scores in Wistar rats with damaged skin after topical application of minoxidil and Gel@MNs for 7 days (*n* = 6). Data are from 3 experiments and presented as means ± SD.

First, we extracted dermal papilla cells with growth function from young mice and verified their purity via their surface marker-Vimentin (Figure 1A). More than 90% of the cells were positively expressed. Next, mDPCs-EV was extracted with ultracentrifugation, and EV surface markers such as CD63, Tsg101, and Alix were expressed (Figure 1B).

The preparation of lipid nanoparticles via the thin film dispersion method [37] has become very extensive. In order to obtain the liposome entrapped with minoxidil, 1 mg of DSPE-PEG_2000_-NHS, 14 mg of Lecithin, 4 μL of Tween-80, and 5 mg of minoxidil were dissolved in 5 mL of absolute ethanol, and a layer of liposome film attached to the inner surface of the flask was obtained via rotary evaporation. Through water bath ultrasound, minoxidil was entrapped in the middle of the liposome to obtain NHS-PEG-Lio @ M. The mDPCs-EV was fully mixed and extruded to obtain MNs with uniform particle size (Figure 1C). Compared with the EV size of 93.59 ± 14.03 nm and the non-encapsulated minoxidil Ns size of 147.2 ± 22.07 nm, the hydrated particle size of MNs showed a small increase, reaching 171.2 ± 25.78 nm (Figure 1E). The Zeta potential showed a negative value, and the absolute value was greater than 25 mV (Figure 1D), indicating the stability of MNs, which also made the drug last longer in the treatment of skin penetration. In order to evaluate the morphology of MNs, we used a transmission electron microscope for detection, and the results showed a spherical shape with uniform characteristics of the membrane structure (Figure 1F). The mixing of gel and MNs was carried out according to the previous method [38] and was confirmed to have porous properties (Figure 1G) (Supplementary Figure S1B).

Next, we set to assess the encapsulation efficiency (EE) and the drug loading (DL) of MNs. In order to find the maximum absorption of minoxidil in the UV spectrum, we first scanned the spectrum at 200 to 400 nm and determined that the maximum absorption wavelength was 231 nm (Supplementary Figure S1A). The results showed that the EE values for minoxidil were 96.40 ± 0.091%, and the DL values were at 20.24 ± 0.015%, respectively (Figure 1H). This indicates that a unit number of EVs can deliver minoxidil more efficiently.

To assess the in vitro safety profile of MNs, their cytotoxic potential was evaluated against murine skin fibroblast cell line L929 and macrophage cell line Raw 264.7 by employing the CCK-8 assay. Subsequent to a 24 h co-incubation period with minoxidil, Ns, and MNs, no discernible alteration in cellular viability was observed when compared to the untreated PBS groups, thereby indicating an excellent in vitro biocompatibility (Supplementary Figure S1C,D). These findings suggest that the developed MNs exhibit negligible cytotoxicity towards both fibroblasts and macrophages, which are key cellular components of the skin and immune system, respectively. The favorable in vitro safety profile demonstrated herein underscores the potential of MNs for further in vivo investigations and future clinical translation.

Skin irritation has become a major obstacle for many drugs to address before clinical research. In order to further detect the skin safety of nanoparticles, we applied minoxidil and Gel@MNs to Wistar rats with skin damage and evaluated the redness and swelling through the skin state. Minoxidil and Gel@MNs did not show inhibitory effect on the self-healing of skin for 7 days (Figure 1I) (Supplementary Figure S2).

### 3.2. Hair Regrowth Evaluation

**Figure 2:**
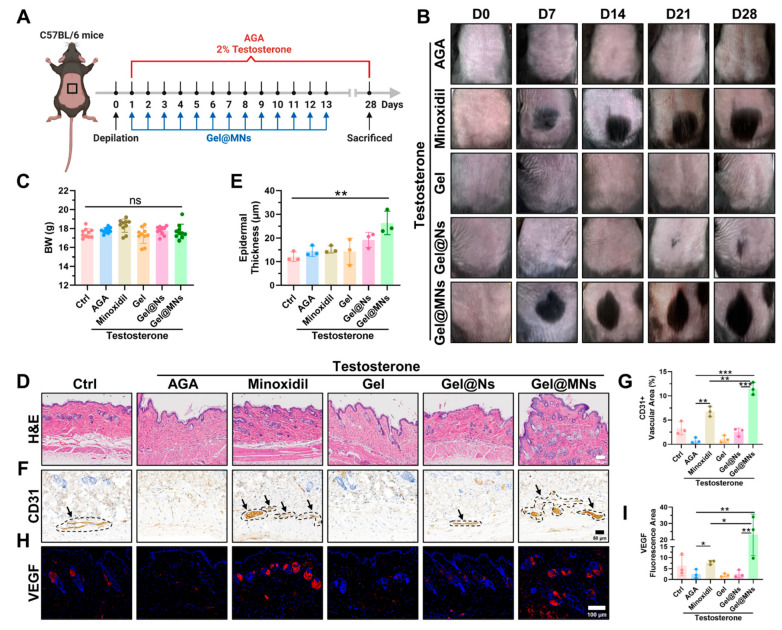
Gel@MNs alleviated testosterone-induced androgenetic alopecia (AGA). (**A**) Schematic representation of the establishment of the AGA mouse model via topical application of testosterone solution daily for 28 days and the therapeutic strategies in the established model. In the model group, only testosterone solution was applied. In the Gel, Gel@Ns, and Gel@MNs groups, nano preparations were applied 13 times once a day. In the minoxidil group, minoxidil was applied daily for 13 days. During the experiment, the hair growth on the back of the mice was recorded weekly (**B**) (*n* = 3). On day 28, the mice were weighed (**C**) (*n* = 10) and euthanized for further analysis. Representative mouse dorsal skin tissues from each group were subjected to H&E staining (**D**) (*n* = 3), and epidermal thickness was quantified (**E**) (*n* = 3). In addition, immunohistochemical staining for CD31 (**F**) (*n* = 3) and immunofluorescent staining for VEGF (**H**) (*n* = 3) were performed on the skin tissues (Among them, the area shown by the arrow is CD31 positive.), and these results were subsequently quantified (**G**,**I**) (*n* = 3). Data are presented as means ± SD. * *p* < 0.05, ** *p* < 0.01, *** *p* < 0.001.

To estimate the hair regrowth efficacy of Gel@MNs, an AGA mouse model was established, followed by different treatments (Figure 2A). The photographs of hair regrowth status over time showed that no obvious hair regrowth occurred in the model group until day 28 (Figure 2B), indicating the successful establishment of the AGA model. Gel@MNs achieved better regenerated hair coverage and hair density (Supplementary Figure S1F) on day 28 than minoxidil. The Gel group did not add mixed nano-lipid particles, and the germinal effect was not obvious, similar to the AGA group. Gel@Ns grew new hair on day 21, because mDPCs-EV targeted dermal papilla cells and the active ingredients promoted the cell cycle from the telogen to the growth phase.

It can be observed from the H&E sections of the skin that small hair follicles were distributed in the dermis of normal mice but disappeared in the AGA group and the Gel group. The minoxidil group showed denser and larger hair follicle units. When the hair follicle becomes larger, it may provide more space and nutrient supply, thereby promoting hair growth. Larger hair follicles can accommodate more cells and hair follicle stem cells, thereby increasing the formation and growth of new hairs. In addition, the increase in hair follicles may also be related to the health and quality of hair. Larger hair follicles mean a healthier environment for hair growth, resulting in stronger, shinier hair. This feature was more pronounced in the Gel@MNs group, and the exosomes doped in the Gel@Ns also slightly increased the growth of some hair follicles (Figure 2D). In addition, we also quantified the thickness of the epidermis between the groups and found that the Gel@Ns group and the Gel@MNs group were the most obvious (Figure 2E). Both groups contained EVs that could target mDPCs, so that the hair follicles were transformed from the telogen phase to the grown phase, and the hair follicle cells with stronger vitality secreted more growth factors, which may also be the reason for the thickening of the epidermis. CD31 is a vascular endothelial cell marker. Immunohistochemical staining showed that the Gel@MNs group had more positive expression, showing the proliferation of blood vessels around the hair follicles, which could provide more oxygen, nutrients and growth factors to support the growth and health of hair (Figure 2F,G). It has been reported that angiogenesis can accelerate anagen induction and increase the diameter of HFs and hairs shafts. As an essential factor for promoting angiogenesis, the level of VEGF in the alopecia area over a long period was evaluated. Compared with the model group, the VEGF level in the skin tissues of the Gel@MNs group was significantly elevated, while there was no significant difference between the Gel group and AGA group (Figure 2H,I). Collectively, we speculated that after targeting mDPCs, Gel@MNs increased the number and volume of hair follicles and the formation of surrounding micro vessels under the dual effects of the precise release of minoxidil and EV and changed the cell cycle.

### 3.3. Gel@MNs Exhibits No Significant Tissue Toxicity

**Figure 3:**
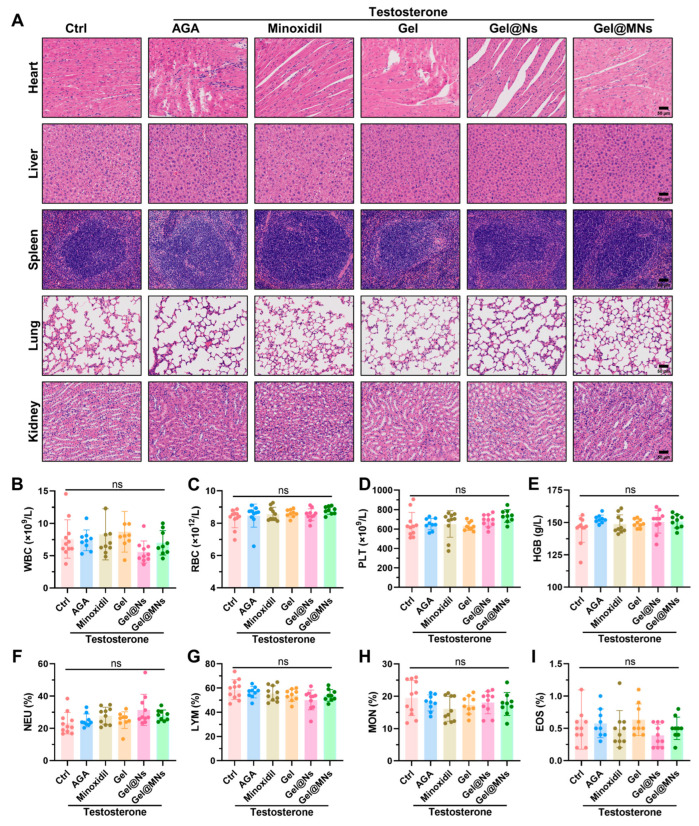
Gel@MNs exhibits excellent biocompatibility during treating mice. The AGA mice model was established and subjected to the naontherapies as described in Figure 2A. (**A**) H&E staining of major organs (heart, liver, spleen, lung, and kidney) (Scale bar = 50 μm) (*n* = 3). (**B**–**I**) Complete blood analysis for blood cell counts (HBG, PLT, RBC, and WBC) and proportions of immune cells (NEU, LYM, MON, and EOS) (*n* = 10). Data are presented as means ± SD. ns, not significant.

In order to evaluate the tissue toxicity of Gel@MNs and whether it will cause an immune response, we collected the blood and organs of each group of mice on day 28. Indeed, H&E staining of tissue slices revealed that no significant tissue damages were induced by the nanoparticles in major organs including the heart, liver, spleen, lung, and kidney (Figure 3A). A complete blood count indicated that there were no marked alterations in the counts of white blood cells (WBCs), red blood cells (RBCs), and platelets (PLTs), as well as the level of hemoglobin (HGB) in mice receiving the nanotherapeutics (Figure 3B–E). Also, the nanoparticle treatment caused no significant effects on the levels of neutrophil (NEU), monocyte (MON), eosinophil (EOS), and lymphocyte (LYM). There was no difference in the proportion of these immune cells. (Figure 3F–I). Thus, our data indicated that Gel@MNs and the associated nanomaterials displayed good biocompatibility with no significant tissue toxicity.

To evaluate the effects of different treatments on skin allergy symptoms in mice, we determined the levels of IgE in skin tissue samples from each group via an enzyme-linked immunosorbent assay (ELISA) after the completion of the treatment cycle. IgE is a critical mediator of allergic diseases, and its local levels in the skin directly reflect the severity of allergic responses. The results demonstrated no significant differences in skin IgE levels among the groups, with no manifestation of allergic phenomena, further validating the in vivo safety of Gel@MNs (Supplementary Figure S1E).

### 3.4. The Regulation of Skin Transcriptome in AGA Mice by Gel@MNs

To explore the mechanism underlying Gel@MNs activity in AGA treatment, we analyzed the transcriptome sequencing (RNA-seq) results of mice skin with or without treatment. Heat map analysis of all of the DEGs based on RNA-seq assay revealed that the gene expression profiles of the AGA group and Gel@MNs group were distinct (Supplementary Figure S3).

**Figure 4:**
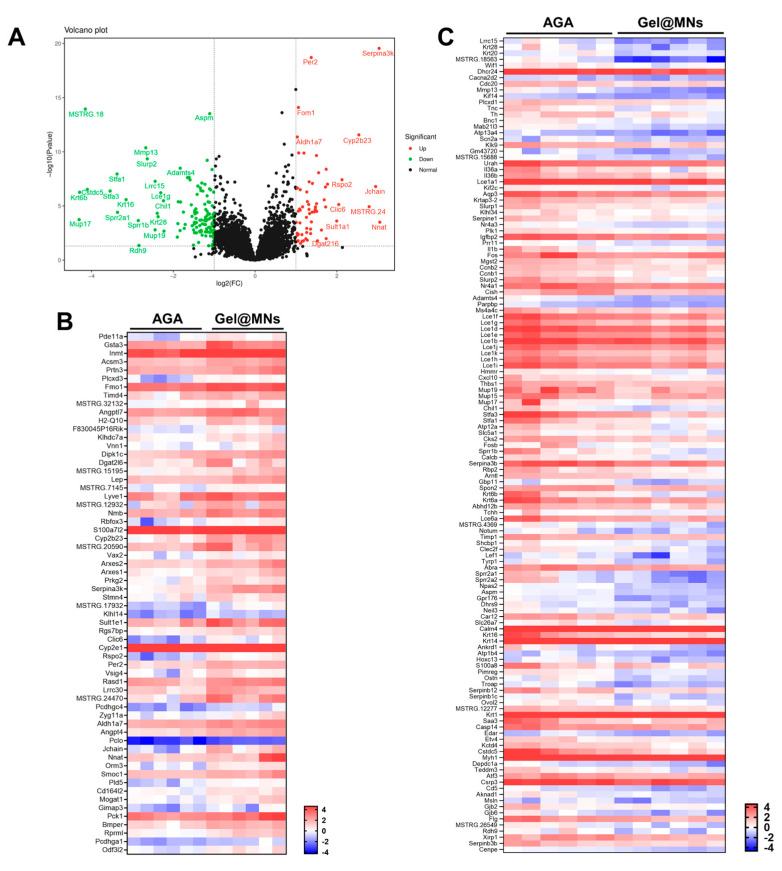
RNA-seq analysis of AGA mice treated with Gel@MNs. (**A**) Volcano plot analyses of total differentially expressed genes (DEGs) between the Gel@MNs treatment group and the model group. Green and red dots represent downregulated and upregulated genes. Grey dots represent the genes not statistically different. Then, heatmap analysis of all upregulated (**B**) and downregulated (**C**) genes was presented.

Firstly, in the volcano plot of all genes between the AGA group and Gel@MNs group (Figure 4A), upregulated and downregulated genes are shown in red and green, respectively. It includes 62 upregulated genes and 133 downregulated genes, and the FPKM value of each sample is shown in the form of a heat map (Figure 4B,C).

Next, we performed gene ontology (GO), Kyoto Encyclopedia of Genes and Genomes (KEGG) analysis, and Gene Set Enrichment Analysis (GSEA) on all differentially expressed genes.

**Figure 5:**
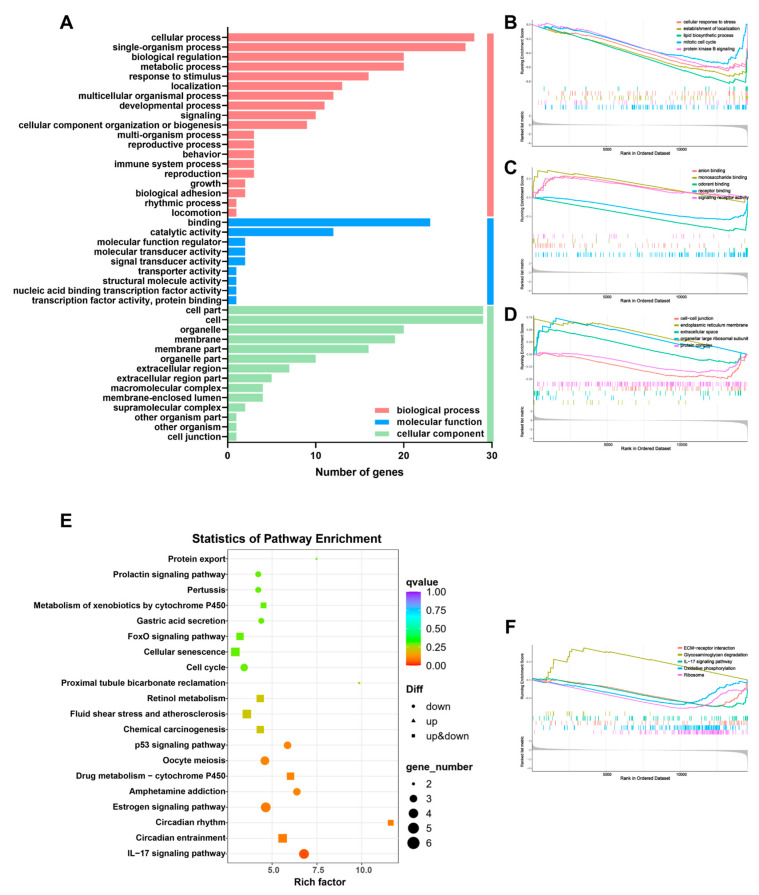
RNA-seq analysis of AGA mice treated with Gel@MNs. GO analysis (**A**) and KEGG analysis (**E**) of model group and Gel@MNs group. GSEA analysis of model group and Gel@MNs group in biological process-related genes (**B**), molecular function-related genes (**C**) and cellular component-related genes (**D**). (**F**) GSEA analysis of model group and Gel@MNs group in KEGG pathway enrichment.

Through GO analysis, it is found that the biggest difference among the three categories is biological process (BP), followed by cellular component (CC), and the smallest difference is molecular function (MF). The cellular process related to BP is significantly different, and it has been reported that it plays a key role in hair follicle and hair growth, such as through cell proliferation, cell differentiation, cell cycle regulation, etc., and the comprehensive regulatory role of a single organism process in the entire hair system, including the interaction of cells around the hair follicle, dermal papilla, blood vessels, and immune cells. The differences in the assembly of the skin cytoskeleton and the functional regulation of organelles before and after treatment were reflected in the CC-related cell part and cell (Figure 5A). Further, GSEA analysis was performed on the three major processes in GO analysis. The results showed that the BP-related lipid biosynthetic process was significantly downregulated after Gel@MNs treatment (Figure 5B). This is due to the occurrence of AGA. In the process, the secretion of androgens is strong, resulting in abnormal lipid metabolism, so it is also called seborrheic alopecia, which is alleviated after treatment. The CC-related processes are intertwined. Among them, the endoplasmic reticulum membrane plays an important role in cell metabolism and lipid synthesis. It is upregulated after treatment (Figure 5D), which accelerates the biological function of the endoplasmic reticulum membrane. In addition, if the cells are under stress, it may also lead to the downregulation of the endoplasmic reticulum membrane to reduce protein load and respond to cell stress, which corresponds to the organellar large ribosomal subunit process in charge of protein synthesis. Monosaccharide binding showed upregulation of genes related to glucose metabolism and glucose signal transduction after treatment in MF-related processes (Figure 5C).

Next, it was found that the functional differences between the AGA control and Gel@MNs groups were mainly focused on the “IL-17 signaling” pathway according to the KEGG analysis (Figure 5E). The number of genes in this pathway is the largest, the proportion is the largest, and the difference is the most obvious (Supplementary Figure S4A). The regulation of the IL-17 pathway plays an important role in acute and chronic inflammatory responses. NF-κB, MAPK, and C/EBPs are its downstream pathways. Activation of this pathway can induce the expression of antimicrobial peptides, cytokines, and chemokines. Compared with the AGA group, the skin inflammation decreased after treatment, which also verified the downregulation of cell stress in GO analysis, thereby inhibiting lipid accumulation, promoting glucose metabolism and protein synthesis, and accelerating biological processes. This downregulation is also reflected in the GSEA analysis (Figure 5F). Surprisingly, the estrogen signaling pathway after Gel@MNs treatment was significantly downregulated, which may be related to the feedback effect between androgens. AGA mice offset the negative effects of androgens on hair follicles by increasing estrogen activity or enhancing estrogen signal transduction. Glycosaminoglycan is an important extracellular matrix component. The glycosaminoglycan degradation pathway is upregulated after a single treatment. This may be due to the decreased ability of hair follicle cells in AGA mice to degrade and remodel the extracellular matrix, thereby downregulating glycosaminoglycan degradation to protect hair follicles from external stimuli, damage, or androgen. It is well known that staying up late will accelerate the secretion of human skin oil, hair loss, and affect the basic metabolism of organisms. Studies have shown that long-term circadian rhythm disorders affect the expression of circadian clock pathway proteins and are associated with changes in the characteristics of epidermal keratinocytes between human hair follicles, human hair follicle keratinocytes, and human hair follicle dermal papilla stem cells. Circadian rhythm is a biological process of endogenous oscillation widely existing in organisms. It is reported that circadian rhythm changes affect the hair growth cycle. Circadian clock genes can regulate hair growth by controlling the cell cycle. Here, we found that the circadian rhythm and circadian entrainment-related pathways in the AGA group and Gel@MNs group are significantly different, and further research is needed to further explore this. It provides new challenges for future research. Furthermore, aging-related gene pathways, such as cell cycle, cell senescence, and the p53 signaling pathway, were downregulated after treatment, which provided strong evidence for the activation of mDPCs.

Through COG and eggNOG Function Classification of Consensus Sequence, we also found that the genes related to post-translational modification, protein turnover, and chaperones occupy the highest value among these differential genes. Gel@MNs may have an impact on the post-translational modification process of proteins, resulting in changes in protein modification patterns. For example, it causes the increase in phosphorylation, acetylation, methylation, and other modifications. In addition, signal transduction mechanisms indicate that the treatment has an effect on the intracellular signal transduction mechanism and regulates cell proliferation, differentiation, apoptosis, or other biological processes (Supplementary Figure S4B,C).

## 4. Discussion

Minoxidil has become the first choice for the treatment of AGA in the contemporary era; it is mainly circulated in the market in the form of sprays and is accepted by a majority of patients, with a very good hair growth effect [39,40]. However, some people may experience a series of side effects before the arrival of the germinal effect and will experience discomfort symptoms such as scalp stimulation, redness, itching, and dryness. More severe cases may sometimes cause systemic adverse reactions, such as hypotension, palpitations, and arrhythmia. Here, we used a mixed lipid nanoparticle that can specifically target mDPCs to achieve precise delivery of minoxidil molecules, so as to reduce a part of the ineffective dosage in the transdermal process, promote the transformation of hair follicle cells with less and safer drugs, and be able to stay in the body and slowly release drugs. Combined with mDPCs, homologous EV can greatly evade the body’s immune response and reduce stimulation. The gel can effectively adhere to the skin and reduce skin dryness.

When Gel@MNs was applied to the skin of AGA mice for 7 days, the hair growth had exceeded the minoxidil group. On day 28, we found that the number of hair follicles in the dermis increased, the volume became larger after treatment, and the expression of CD31 and VEGF increased, indicating that Gel@MNs could not only reawaken the senescent cells of hair follicles in the telogen into young cells with division function and secretion of growth factors, it also increases the formation of micro vessels around hair follicles. Through subsequent transcriptomics studies, we found that the IL-17-related pathway was upregulated in the skin of AGA mice, which was a sign of inflammatory response. It is precisely because of this inflammation that cells produce stress, resulting in a large amount of lipid accumulation, glucose metabolism, and protein synthesis. Blocked nutrient transport decreased, the cell cycle was arrested, circadian rhythm-related gene disorders diminished, cells entered the telogen, and hair follicles shrunk. These phenotypes were greatly alleviated after Gel@MNs treatment. Concurrently, no evident tissue damage and blood toxicity were associated with the treatment.

The innovative design of the Gel@MNs delivery system has brought a novel insight into the treatment of AGA. We found that excessive secretion of testosterone may disrupt the normal function of skin metabolism and cell cycle-related pathways. By precisely delivering Gel@MNs to hair follicle cells in the resting phase, not only can the loaded minoxidil molecules promote the reactivation of these follicular cells, but the mDPCs-EVs themselves can also rejuvenate the dermal papilla cells, allowing them to transition from the resting state to the growth phase. Therefore, Gel@MNs represents a novel paradigm for targeted AGA therapy, exhibiting superior therapeutic efficacy, safety, and controlled release properties compared to traditional formulations.

Furthermore, the molecular mechanisms by which EVs promote the activation of DPCs remain insufficiently understood. In future research, we can employ in vitro cellular experiments combined with multidisciplinary omics techniques, such as transcriptomics, proteomics, and metabolomics, to deeply investigate the novel regulatory mechanisms underlying EV-mediated DPC activation. This will provide a theoretical foundation for further optimization and development of relevant therapeutic strategies. 

It is noteworthy that nanodrug delivery systems have made significant strides and are widely applied in clinical oncology. For instance, liposomal formulations of doxorubicin and nanoparticle albumin-bound paclitaxel have demonstrated exceptional efficacy and safety profiles. This provides a strong reference for developing novel nanotherapeutics for the treatment of AGA. Currently, with the accelerated pace of life, increased stress levels, and the prevalence of unhealthy habits such as sleep deprivation, AGA has become increasingly common among younger populations, imposing a significant burden on their mental health and quality of life. Therefore, there is an urgent need to develop more effective, safe, and long-term AGA treatment strategies. In the future, it is hoped that this scheme of combining DPCs and EVs with hair growth promoters such as minoxidil can be recognized by the public. This approach aims to maximize the bioactive regulatory effects of EVs and the hair growth-promoting effects of minoxidil, offering AGA patients a novel treatment option.

## 5. Conclusions

Gel@MNs nano preparations have shown promising potential in the treatment of AGA. By accurately delivering minoxidil molecules to DPCs, Gel@MNs can reduce ineffective doses, promote reactivation of hair follicle cells and the secretion of growth factors at less and safer drug doses, and enhance the formation of micro vessels around hair follicles. This innovative drug delivery system not only significantly improves the efficacy but also reduces the risk of systemic adverse reactions.

## Data Availability

The data presented in this study are available upon request from the corresponding author.

## Supplementary Materials

The following supporting information can be downloaded at: https://www.mdpi.com/article/10.3390/pharmaceutics16070935/s1, Figure S1: (A) UV absorption spectra of the vehicle and minoxidil in the range of 200 to 400 nm. (B) The final products of the preparation process are blank Gel and Gel containing MNs. L929 cells (C) and Raw 264.7 cells (D) were incubated with PBS, Minoxidil (5 μg/mL), Ns (20 μg/mL) and MNs (25 μg/mL) for 24 h. Then Cell viability was detected. (*n* = 3) (E) The content of lgE in the skin of mice in each group was detected after the end of the treatment cycle by Elisa. (F) Amplified display of hair growth on the dorsal area of AGA mice treated with minoxidil and nano preparations. Data are presented as means ± SD. * *p* < 0.05, ** *p* < 0.01, *** *p* < 0.001, **** *p* < 0.0001; Figure S2. (A) Photographs were taken to display the skin redness and swelling conditions in Wistar rats with damaged skin after topical application of Minoxidil and Gel@MNs for 7 days. Assessment was performed according to the criteria for skin redness and swelling score (B); Figure S3. Clustering heatmap of all differential gene expression patterns between the Gel@MNs treatment group and the model group; Figure S4. (A) A 10 × 10 dot plot was generated to visualize the number of genes in differentially enriched pathways between the Gel@MNs treatment group and the model group in the KEGG pathway enrichment analysis. The differential expression gene eggNOG (B) and COG (C) annotation classification statistics were presented in a graph comparing the Gel@MNs treatment group and the model group.
