# Supplementary material for: Engineered Exosomes Biopotentiated Hydrogel Promote Hair Follicle Growth via Reprogramming the Perifollicular Microenvironment
Source: Pharmaceutics. 2024 Jul 13;16(7):935. doi: 10.3390/pharmaceutics16070935 (PMC11279965; doi:10.3390/pharmaceutics16070935)
Supplement: Supplementary file 1 [file pharmaceutics-16-00935-s001.zip › pharmaceutics-3047100-supplementary.pdf]

Supplementary Materials: Engineered Exosomes Biopotentiated Hydrogel Promote Hair Follicle Growth via Reprogramming the Perifollicular Microenvironment

Hairui Zhang, Jiali Yao, Qianyang Jiang, Yurou Shi, Weihong Ge and Xiaoling Xu

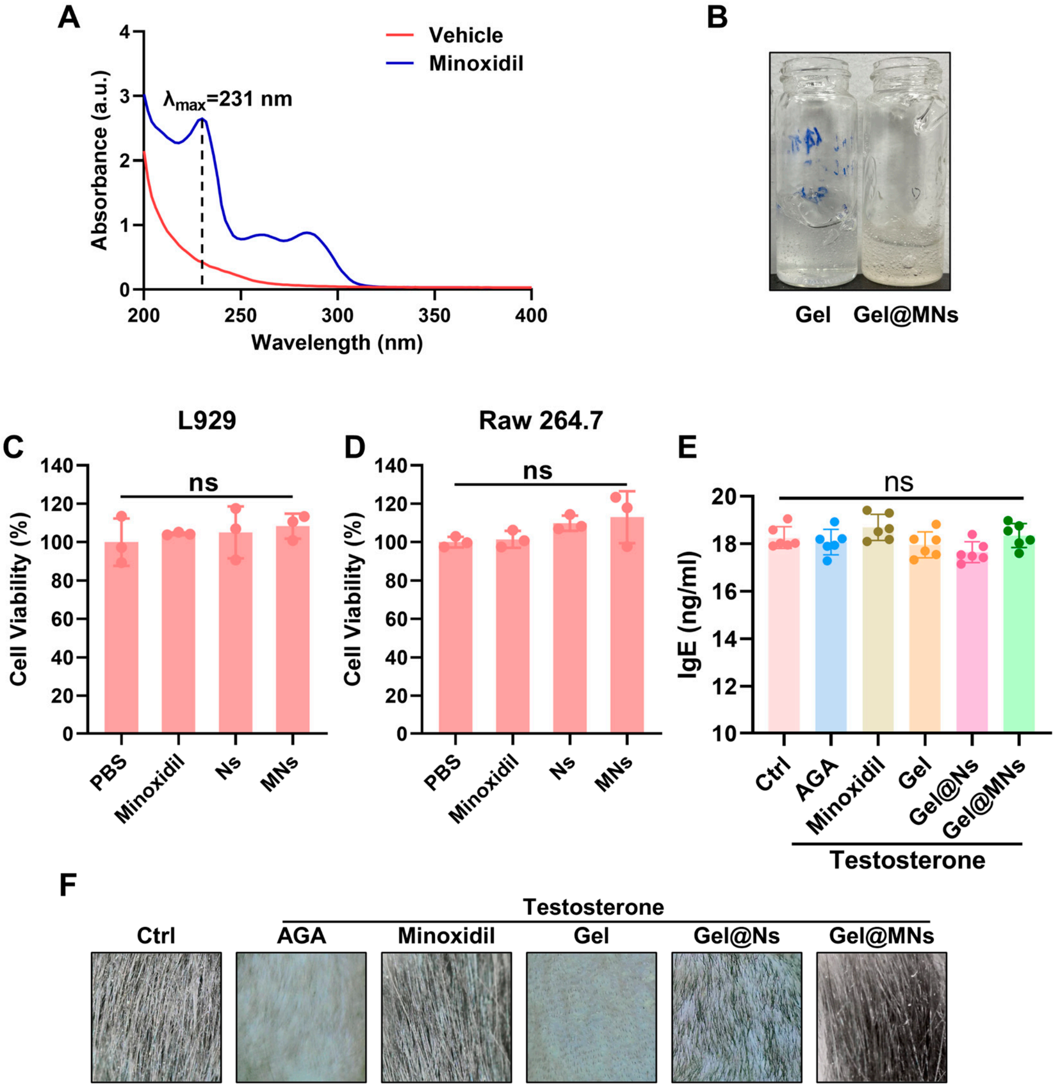

Figure S1. (A) UV absorption spectra of the vehicle and minoxidil in the range of 200 to 400 nm. **(B)** The final products of the preparation process are blank Gel and Gel containing MNs. L929 cells **(C)** and Raw 264.7 cells **(D)** were incubated with PBS, Minoxidil (5 μg/ml), Ns (20 μg/ml) and MNs (25 μg/ml) for 24 h. Then Cell viability was detected. (n= 3) **(E)** The content of IgE in the skin of mice in each group was detected after the end of the treatment cycle by Elisa. **(F)** Amplified display of hair growth on the dorsal area of AGA mice treated with minoxidil and nano preparations. Data are presented as means ± SD. ns, not significant.

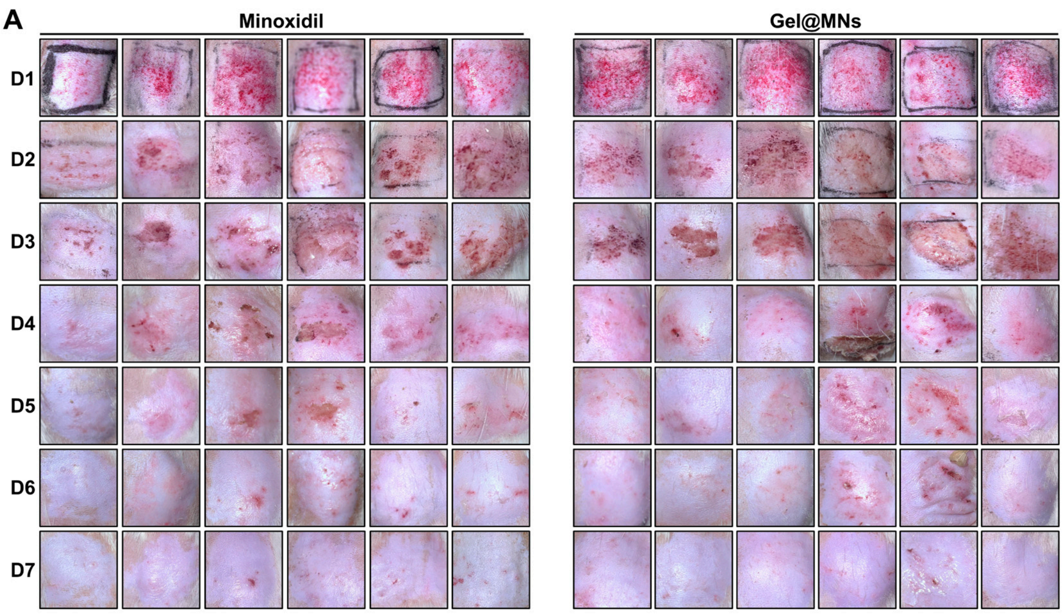

B

Redness Score	Symptom	Swelling Score	Symptom
0	Normal	0	Normal
1	Mild	1	Mild
2	Obvious	2	Obvious
3	Severe	3	Severe (1mm skin bulge)
4	Purple red erythema with eschar	4	The skin bulge is more than 1mm. And there is expansion

Figure S2. (A) Photographs were taken to display the skin redness and swelling conditions in Wistar rats with damaged skin after topical application of Minoxidil and Gel@MNs for 7 days. Assessment was performed according to the criteria for skin redness and swelling score **(B)**.

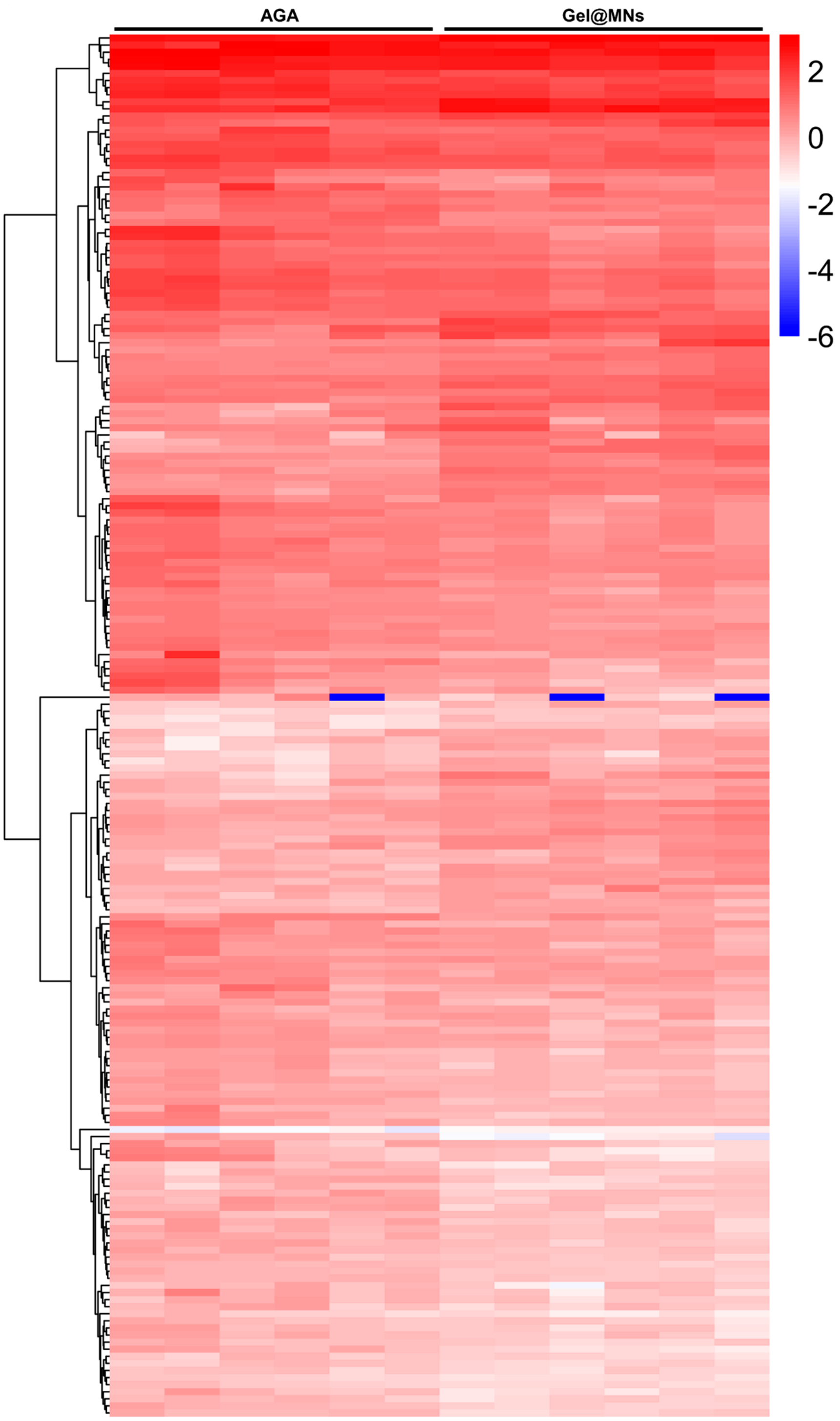

Figure S3. Clustering heatmap of all differential gene expression patterns between the Gel@MNs treatment group and the model group.

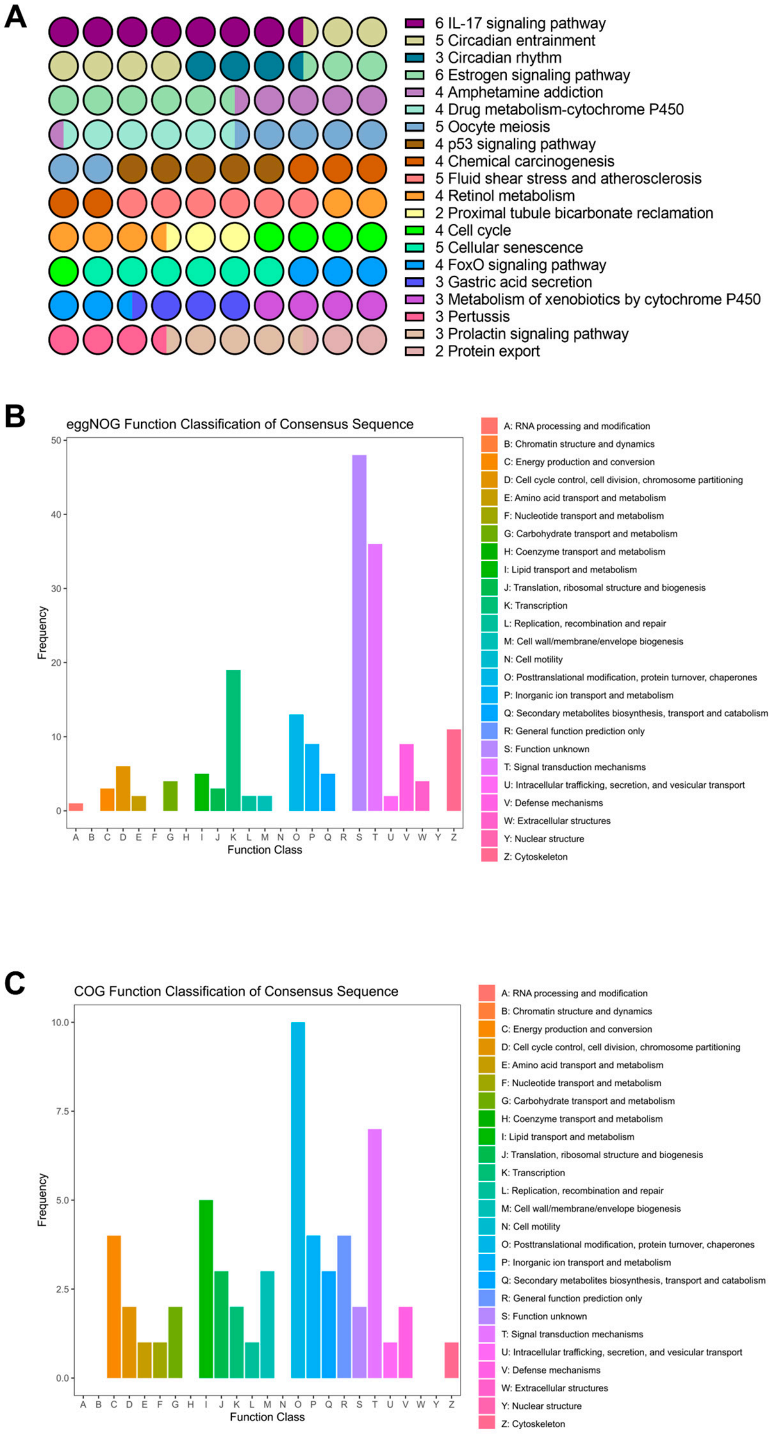

Figure S4. (A) A 10x10 dot plot was generated to visualize the number of genes in differentially enriched pathways between the Gel@MNs treatment group and the model group in the KEGG pathway enrichment analysis. The differential expression gene eggNOG **(B)** and COG **(C)** annotation classification statistics were presented in a graph comparing the Gel@MNs treatment group and the model group.
